# Assessing Biodiversity at Eastern Oyster ( *Crassostrea virginica* ) Aquaculture and Reef Sites Utilizing Real‐Time Monitoring and Environmental DNA in Rehoboth Bay, Delaware, USA

**DOI:** 10.1002/ece3.72450

**Published:** 2025-11-09

**Authors:** Tahera Attarwala, Ali Parsaeimehr, Gulnihal Ozbay

**Affiliations:** ^1^ Department of Agriculture and Natural Resources, College of Agriculture, Science, and Technology Delaware State University Dover Delaware USA; ^2^ Department of Biology and Microbiology, College of Natural Sciences South Dakota State University Brookings South Dakota USA

**Keywords:** biodiversity, eastern oyster, environmental DNA, real‐time monitoring, Rehoboth Bay

## Abstract

Eastern Oysters ( *Crassostrea virginica* ) are a keystone species and an important product of the commercial shellfish industry in Delaware. Oysters are known as “environmental engineers” that provide a structured habitat for the ecosystem, thus promoting biodiversity. In order to further investigate the role oysters play in increasing biodiversity, real‐time monitoring and environmental DNA (eDNA) were conducted at different sites around Rehoboth Bay, Delaware, USA. The sites include pilot artificial reefs, private aquaculture farms, and a control site without any oysters or habitat structure. Underwater GoPro Hero 3+ and 8 cameras were deployed every 2 weeks from June to October in 2022 and 2023. Cameras were deployed for approximately 2–3 h at a time, and upon retrieval, cameras were reviewed for any signs of aquatic life, and all documented species were identified and recorded for comparisons between sampling sites. Water samples were collected simultaneously for eDNA analysis to serve as a complementary method for species identification. DNA isolation and polymerase chain reaction (PCR) were performed to amplify gene sequences for targeted species. Using camera technology, 23 different animal species were recorded across the five study sites. The most abundant species included Spot ( *Leiostomus xanthurus* ), Atlantic Silverside ( *Menidia menidia* ), Atlantic Menhaden ( *Brevoortia tyrannus* ), Horseshoe Crab *(Limulus polyphemus*), Blue Crab ( *Callinectes sapidus* ), and Hermit Crabs ( *Pagurus longicarpus* ). The eDNA analysis also successfully detected these species, highlighting the effectiveness of eDNA as a tool for species monitoring. Notably, there was considerable overlap between species identified through both real‐time monitoring and eDNA methods. These findings contribute to ongoing oyster restoration initiatives and sustainable aquaculture practices in the Delaware Inland Bays (DIB), while also enhancing our understanding of complementary biodiversity monitoring techniques.

## Graphical abstract

Eastern oysters ( *Crassostrea virginica* ) are a keystone species and vital to the commercial shellfish industry, acting as environmental engineers that enhance biodiversity. To study their impact, real‐time monitoring and Environmental DNA analysis were conducted at various sites in Rehoboth Bay, Delaware and 23 species were identified, with Spot, Atlantic Silverside, and Blue Crabs among the most abundant. The findings highlight the role of oysters in supporting marine life and contribute to ongoing oyster restoration and sustainable aquaculture efforts in the Delaware Inland Bays.

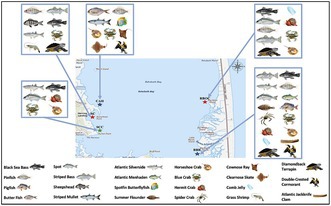

## 1 Introduction

Rehoboth Bay (38.6610° N, 75.0962° W) is one of three systems that comprise the Delaware Inland Bays (DIB) and is located in the southernmost part of the state (Berman et al. 2013). The land use is designated predominantly for residential and recreational development (Hauser and Bason 2020). This area attracts both seasonal visitors and permanent residents each year and continues to grow in both population size and economic value. The DIB generates over 4.5 billion annual income and supports the creation of over 35,000 jobs. Oyster aquaculture is one of the important re‐emerging industries in the state that contributes over a million dollars to the annual income of the Inland Bays (Hauser and Bason 2020). Eastern oysters ( *Crassostrea virginica* ) are keystone species that provide many ecosystem services including water filtration, nutrient removal, and shoreline protection (Ewart 2013; Raj 2008; Coen et al. 2007). Oyster reefs and aquaculture gear can provide habitat structure and foraging grounds for many different animal species which promotes overall biodiversity (Ozbay et al. 2013; Martínez‐Baena et al. 2022; Bishop et al. 2023). Delaware Bay supports a variety of species including over 30 species of shorebirds and the largest population of Horseshoe Crabs in North America (Anstead et al. 2023). The study of species distribution and richness in the aquatic environment is essential for population management and conservation (Tsuji et al. 2018) and understanding the role oysters have in promoting biodiversity can foster restoration efforts and legislation to protect the current oyster populations (Ozbay et al. 2013).

There are several methods to monitor biodiversity in the aquatic environment including the use of camera technology for real‐time monitoring. The use of real‐time monitoring has been applied in numerous research areas because it is cost‐effective, easy to deploy, provides minimal disturbance to the environment, and generates a permanent archive that can be referenced later on (Zarco‐Perello and Enríquez 2019; Mercaldo‐Allen et al. 2021). Several studies have demonstrated the capabilities of using camera technology for biomonitoring (Wall et al. 2014). For example, underwater cameras were used to observe the interactions between fish and crab species near oyster aquaculture sites (Ferriss et al. 2021; Shinn et al. 2021). However, camera‐based studies inherently face limitations, such as restricted field of view and reduced effectiveness under low‐visibility conditions (Ferriss et al. 2021). To address these challenges and broaden the scope of ecological assessments, the integration of complementary monitoring techniques is essential. One such emerging method is environmental DNA (eDNA), an innovative tool for detecting and identifying species in both terrestrial and aquatic environments, offering enhanced sensitivity and noninvasive sampling capabilities. eDNA is defined as organismal DNA or genetic material that can be collected through soil and water samples and can detect and identify species in the environment (Taberlet et al. 2021; Thomsen et al. 2012; Thomsen and Willerslev 2015; O'Donnell et al. 2017; Liu et al. 2019; Ruppert et al. 2019). eDNA has the potential to detect species that may not be observed through traditional monitoring methods such as field observations, visual surveys, and catch and release (Civade et al. 2016; Zou et al. 2020; Cole et al. 2022). There are several studies that have begun incorporating eDNA for biomonitoring (Coutts et al. 2022; Tsuji et al. 2018; Valentini et al. 2016; Rees et al. 2014). In one study, real‐time polymerase chain reaction (commonly referred to as qPCR) was used to successfully detect multiple species from eDNA samples using species‐specific primers (Tsuji et al. 2018). In another study, eDNA was used to inform restoration decisions of an extinct oyster species and demonstrated the ability of eDNA to detect rare species and aid in future restoration efforts (Coutts et al. 2022). There have been studies that aimed to employ both video and eDNA methods to assess biodiversity and habitat function of oyster sites (Clark et al. 2024; Mercaldo‐Allen et al. 2021; Jeunen et al. 2020). For example, Mercaldo‐Allen et al. (2021) observed seven fish species using action cameras but detected 42 fish species through eDNA metabarcoding near oyster aquaculture cages.

In order to expand monitoring efforts, both underwater cameras and eDNA were used in this study. The objectives were to (1) assess biodiversity at *C. virginica* sites in Rehoboth Bay, Delaware, USA using underwater cameras for real‐time monitoring, and (2) introduce eDNA analysis as a complementary method for detecting species presence. The overall goal of this project is to help promote sustainable aquaculture and continued reef restoration efforts to enhance oyster populations and ecosystem functionality throughout the DIB.

## 2 Materials and Methods

### 2.1 Study Sites

**FIGURE 1:**
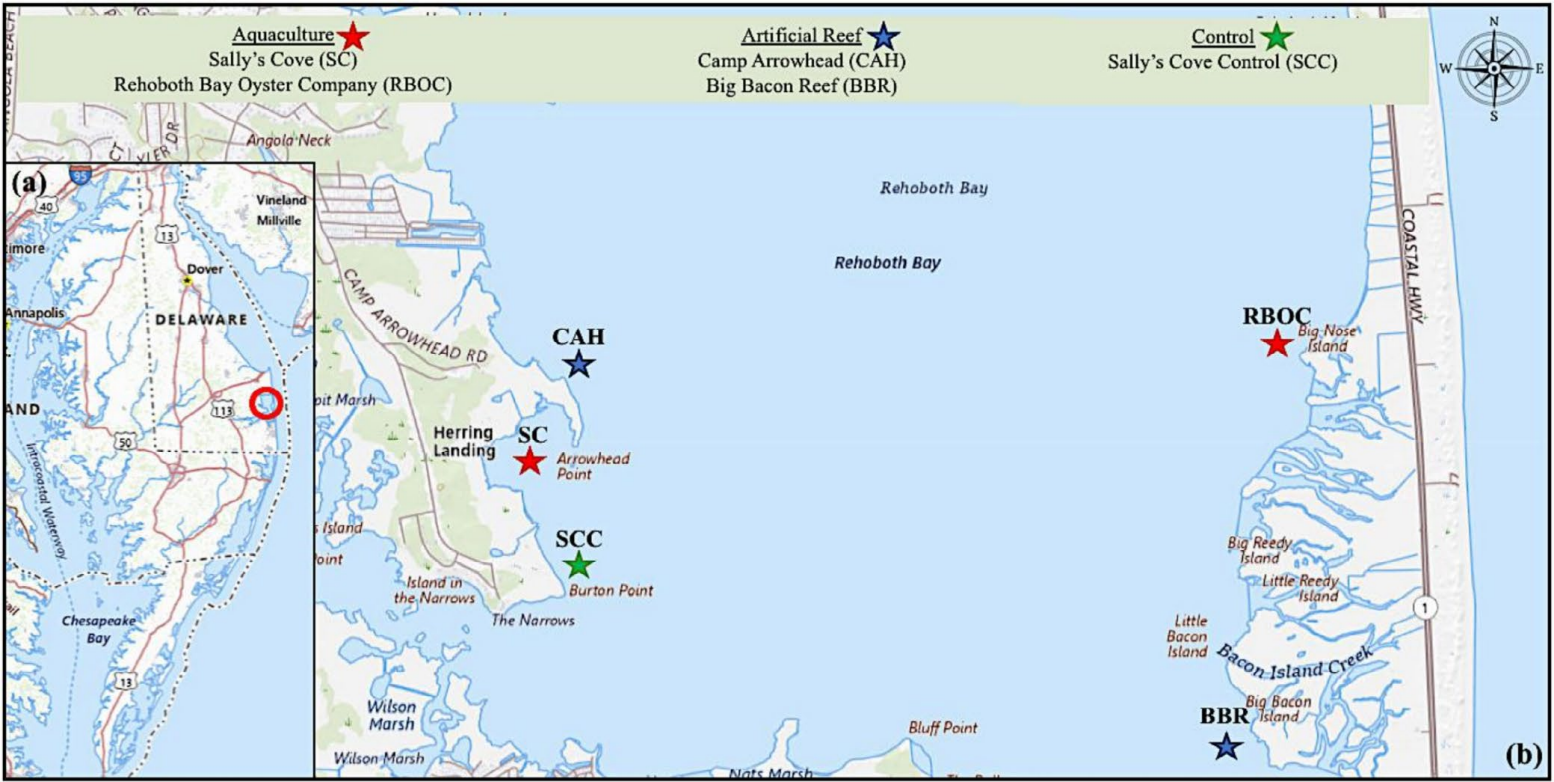
(a, b) Specific locations of study sites where sampling took place. (a) a map of the state of Delaware, USA and (b) the location of study sites in Rehoboth Bay; Sally's Cove (38.64877° N, 75.12870° W), Rehoboth Bay Oyster Company (38.39549° N, 075.04797° W), Camp Arrowhead (38.65430° N, 75.12589° W), Big Bacon Reef (38.38007° N, 075.04866° W), and Sally's Cove Control (38.64446° N, 75.12656° W). The red stars represent on‐going oyster aquaculture, the blue stars represent pilot artificial oyster reefs and the green star represents a natural site with no oysters or structure present.

There was a total of five sites located on both the east and west sides of Rehoboth Bay, Delaware, USA. These sites include two private aquaculture farms, two pilot artificial oyster reefs, and one control site (Table 1 and Figure 1). The aquaculture sites are located at Sally's Cove (SC) and Rehoboth Bay Oyster Company (RBOC). The majority of the cages on the aquaculture farms are off‐bottom cages suspended within the water column, with several cages placed on the sediment bottom. The pilot artificial oyster reefs are located at Camp Arrowhead (CAH) and Big Bacon Reef (BBR). The artificial reefs were created using recycled oyster shells and seeded with farm‐grown oyster seeds by the Delaware Center for Inland Bays. Lastly, the control site is located adjacent to SC and is referred to as Sally's Cove Control (SCC). The control site is located near a sandy beach and is without any oysters or habitat structures. Each site is located up to 500 m from one another. Distance between sites is an important criterion, especially when considering the transport of eDNA in the water.

**TABLE 1:** Description of monitoring sites located in Rehoboth Bay, Delaware, USA, with exact coordinates.

Site name	Site description	Latitude/longitude
Rehoboth Bay Oyster Company	Private aquaculture farm	38.39549° N, 075.04797° W
Sally's Cove	Private aquaculture farm	38.64877° N, 75.12870° W
Big Bacon Reef	Pilot artificial reef	38.38007° N, 075.04866° W
Camp Arrowhead	Pilot artificial reef	38.65430° N, 75.12589° W
Sally's Cove Control	Control site	38.64446° N, 75.12656° W

### 2.2 Real‐Time Monitoring

**FIGURE 2:**
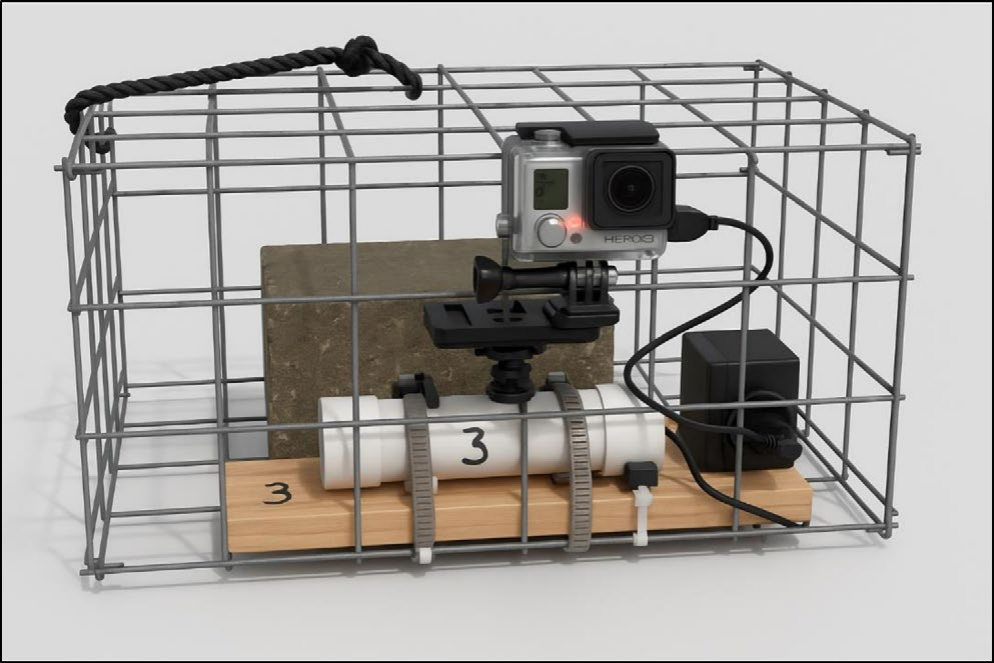
Set‐up and materials used to build the camera housing for real‐time monitoring.

Underwater GoPro Hero 3+ and 8 cameras were deployed for real‐time monitoring. Each camera was secured into a recreational crab trap (Figure 2) using a wood plank (1), PVC pipe (1), Philip screws (3), hex and wing nuts (3), and 4 ½ inch hose clamps (2). Each cage had a concrete brick attached to the inside for added weight and was secured to a float buoy. In order to prolong recording time, a battery extender was also attached to each camera during deployment. There was a total of four cameras used for this study, allowing for monitoring of two sites at a given time with two cameras deployed at each location. Cameras were deployed every 2 weeks from June to October 2022 and 2023. During each sampling week, there would be 2 days when cameras were deployed; the west side was recorded on Day 1 and the east side was recorded on Day 2 of deployment. Cameras were deployed for approximately 2–3 h before retrieval. The footage was then manually reviewed for any signs of life, and all documented species were identified to the closest species level and recorded for comparisons between sampling sites.

### 2.3 Sample Collection and Preparation for eDNA Isolation

Water samples were collected from the mid‐water column at each of the sites using a water depth collector. Different sample size volumes of water were considered but the sample size was increased to 8 L from each site and then transported to the laboratory on ice. In order to prepare the samples for eDNA isolation, the water sample went through a continuous process of filtration and centrifugation until the water was reduced to a volume of around 45 mL including accumulated biomass. The water samples were then stored at −20°C for approximately 2 days as a cold treatment. A cold storage or treatment slows the degradation rate of eDNA (Strickler et al. 2015). The cold treatment improved the overall quality and yield of the eDNA and preserved it for DNA isolation, which is consistent with Hinlo et al. (2017).

### 2.4 Isolation of eDNA

Initially, two eDNA isolation methods were tested: (1) Cetyltrimethyl ammonium bromide (CTAB) method and (2) QIAprep Spin Miniprep Kit (Qiagen Cat. No 27106X4) which was adapted for environmental samples. The different methods were tested using a variation of sample sizes of water (Tables 2 and 3). The QIA Spin Miniprep Kit produced a slightly higher quantity of eDNA and was more cost‐efficient, so it was the selected method for the remainder of the eDNA analysis. At the first stage, the frozen samples were thawed on ice and then centrifuged at 5000 rpm for 15 min. Then the sample was transferred to a 2 mL tube and centrifuged again at 11,000 rpm for 2 min. After centrifuging, the supernatant was removed and 250 μL of nuclease‐free water was added to the sample and vortexed for 30 s. After vortexing, 350 μL of Lysis Buffer was added to the sample and inverted for 1 min, then 350 μL of Neutralization Buffer was added. Next, the sample was again inverted 10 times, stood for 1 min, and then centrifuged at 13,000 rpm for 10 min. Afterwards, the sample was transferred to a spin column tube with a filter that was included with the extraction kit. After transferring, 50 μL of Binding Buffer was added and centrifuged at 12,500 rpm for 30 s. Then the supernatant was discarded; 750 μL of Wash Buffer was added and centrifuged again at 12,500 rpm for 30 s. The supernatant was again discarded and centrifuged at 12,500 rpm for 60 s. Afterwards, the filter was removed and placed into a clean 2 mL tube. Lastly, ~25 μL of nuclease‐free water was added down the middle of the filter for elution. The sample sat for approximately 30 min to 1 h before it was centrifuged at 12,500 rpm for 1 min at 4°C. Once complete, the filter was discarded, and the sample was analyzed using a Nanodrop to determine the quality and quantity of the extracted eDNA.

**TABLE 2:** Quantity and quality of extracted eDNA from the aquatic environment using a CTAB method.

Sample size (L)	Concentration (ng/μL)	260/280 ratio	260/230 ratio
0.5	4 ± 0.4	2.00	3.20
1	10 ± 1.0	1.60	2.50
2	14 ± 1.8	1.85	2.00
4	20 ± 2.8	1.82	2.04

*Note:* Data are presented based on the mean value of three different repeats.

**TABLE 3:** Quantity and quality of extracted eDNA from the aquatic environment using the Qiagen QIAprep Spin Miniprep Kit.

Sample size (L)	Concentration (ng/μL)	260/280 ratio	260/230 ratio
0.5	6 ± 0.5	1.50	2.80
1	12 ± 0.9	1.78	2.10
2	15 ± 1.8	1.80	2.00
4	30 ± 1.5	1.90	2.01

*Note:* Data are presented based on the mean value of three different repeats.

### 2.5 Primer Design

In this initial eDNA analysis, five primers were selected to target three aquatic species. The target genes and corresponding sequences were identified through a review of the scientific literature (Table 4) and verified using resources from the National Center for Biotechnology Information (NCBI) and Primer3Plus. DNA samples were amplified using a 7500 real‐time PCR System, and the presence/absence of the target species was determined using Applied Biosystems 7500 software.

**TABLE 4:** Compilation of the primers identified for PCR analysis along with the corresponding targeted species.

Identifying no.	Specific primer	Targeted species	Sequences	Annealing temperature, °C	References
1	COX1	*Limulus polyphemus*	F:GTATAGCTCACGCAGGAGCCTCA R:GTCAAGTCTACTGAGGCTCCTGC	57°	Lavrov et al. (2000)
2	eef1a‐g2	*Paralichthys dentatus*	F:AGCAGCTCATCGTTGGAGTC R:TGGGGACGAAAGCAACACTT	57°	Caruso (2015)
3	Csap1	*Callinectes sapidus*	F:AAAAATTTGGCGGTGGTTC R:ATTAGATCAAGGTGCAGCTTATG	52°	Lee et al. (2020)
4	SrRNA	*Limulus polyphemus*	F:ATCTGCTCTGTAATCGATGGTCC R:ACGAGGACCATCGATTACAGAGC	57°	Lavrov et al. (2000)
5	eef1a	*Paralichthys dentatus*	F:GCCGAGCGTGAGCGTGGTAT R:ACCCTCCTTGCGCTCAACCT	57°	Caruso (2015)

*Note:* Primers were identified from scientific literature and sequences were cross‐referenced using NCBI and Primer3Plus.

### 2.6 Conditions for Real‐Time PCR

Amplification was performed in a 15 μL reaction volume and consisted of 1 μL of eDNA (concentration of at least 15 ng/μL), 0.5 μL of forward and reverse primer, 7.5 μL of Power SYBR Green PCR Master Mix (Applied Biosystems Cat. No. 4367659), and 6 μL of nuclease‐free water. The negative controls consisted of each of the above‐mentioned solutions with the exclusion of the eDNA. The positive controls were prepared using a minimal‐impact approach with live animals. The animal was incubated for 20–30 min at ambient temperature with minimal aeration, then returned to the original collection site. The incubation water was collected and processed similarly to the other eDNA samples. The mixture was amplified using the 7500 Applied Biosystems software. The samples were programmed for 40 cycles at the following temperature settings: 60 s at 60°C for initial denaturation, followed by 10 min at 95°C for denaturation, 15 s at 95°C for annealing, 60 s at 60°C for extension, and final extension at 60°C for 60 s.

## 3 Results

### 3.1 Species Detected Using Real‐Time Monitoring

**FIGURE 3:**
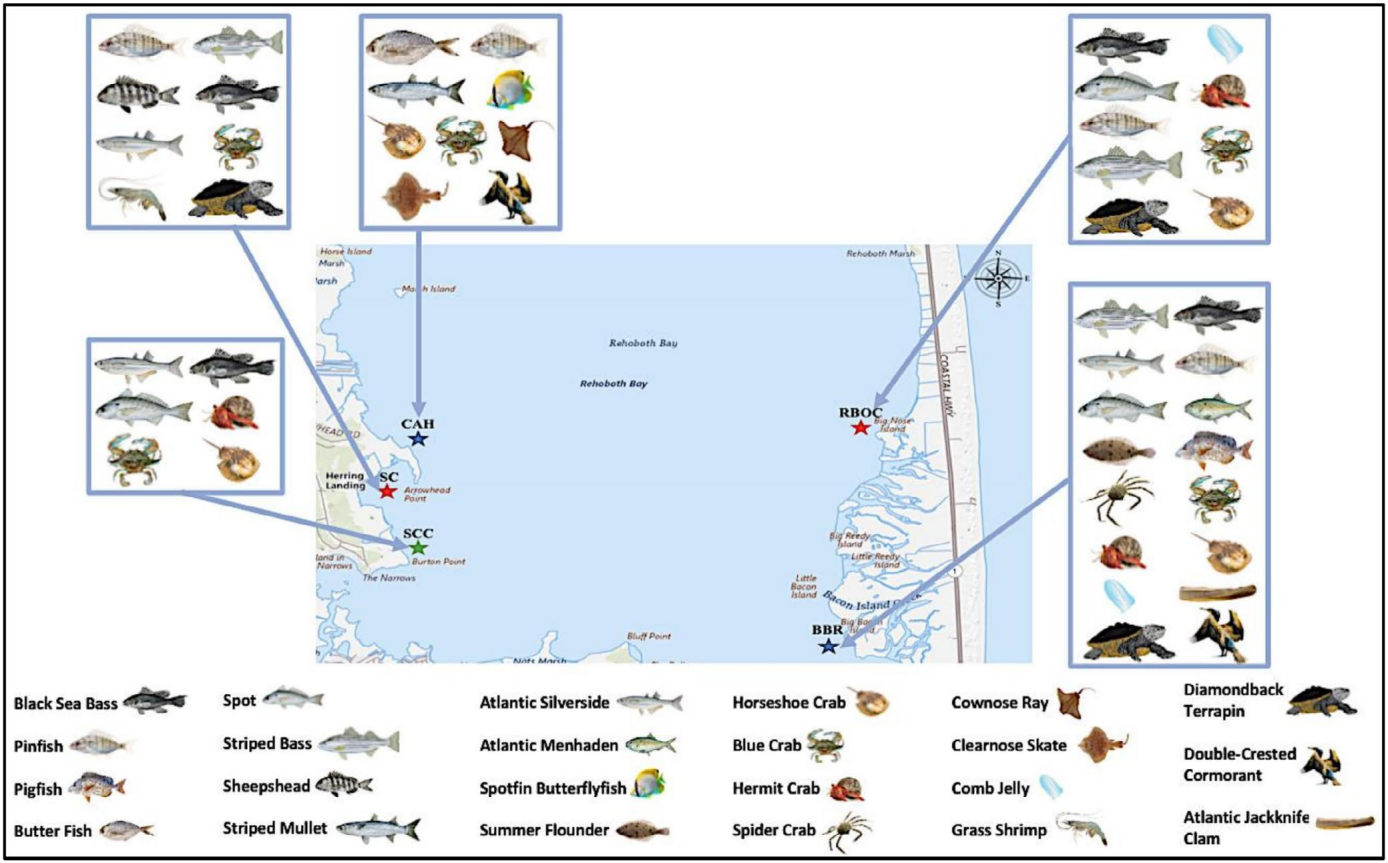
Different species that were detected through real‐time monitoring at each of the study sites. More species were observed on the east side of Rehoboth Bay, Delaware, USA compared to the west.

Cameras were deployed for a total of 20 separate sampling events throughout June to October 2022 and 2023. It is important to note that there were occasions where sampling days needed to be postponed or locations needed to be alternated due to inclement weather. A total of 23 species were observed and identified on the cameras within the 2 years (Figure 3). The species include Cownose Ray ( *Rhinoptera bonasus* ), Clearnose Skate ( *Raja eglanteria* ), Horseshoe Crabs ( *Limulus polyphemus* ), Blue Crabs ( *Callinectes sapidus* ), Hermit Crabs ( *Pagurus longicarpus* ), Spider Crabs ( *Libinia emarginata* ), Spot ( *Leiostomus xanthurus* ), Black Sea Bass ( *Centropristis striata* ), Pinfish ( *Lagodon rhomboides* ), Pigfish ( *Orthopristis chrysoptera* ), Atlantic Silverside ( *Menidia menidia* ), Atlantic Menhaden ( *Brevoortia tyrannus* ), Summer Flounder ( *Paralichthys dentatus* ), Spotfin Butterflyfish ( *Chaetodon ocellatus* ), Butter Fish ( *Peprilus triacanthus* ), Striped Bass ( *Morone saxatilis* ), Sheepshead ( *Archosargus probatocephalus* ), Striped Mullet (Mugil *cephalus* ), Comb Jelly ( *Mnemiopsis leidyi* ), Grass Shrimp ( *Palaemonetes paludosus* ), Atlantic Jackknife Clam (*Ensis leei*), Diamondback Terrapin ( *Malaclemys terrapin* ), and Double‐crested Cormorant ( *Phalacrocorax auritus* ).

**FIGURE 4:**
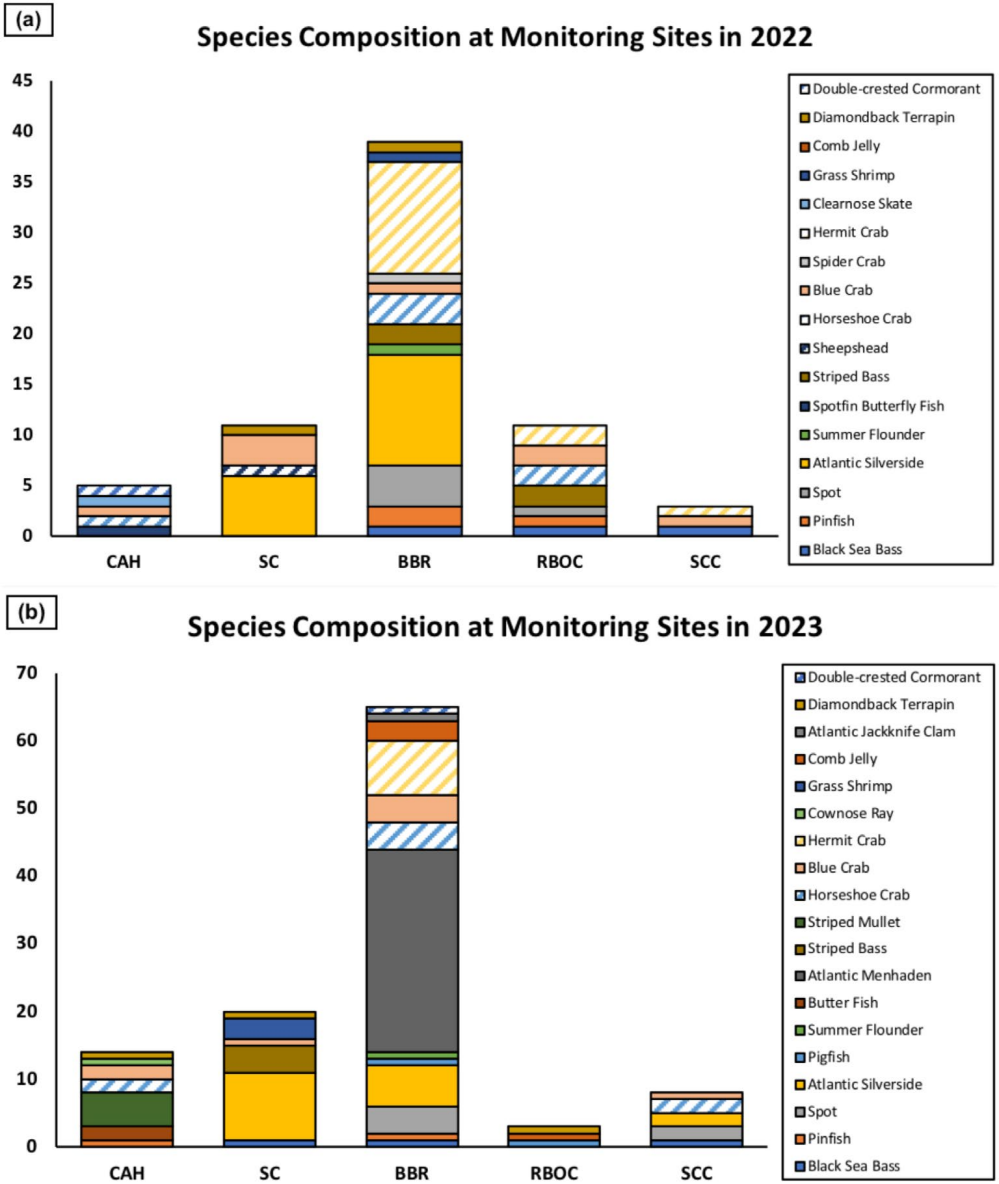
(a, b) Difference in species composition at the monitoring sites using the underwater cameras for real‐time monitoring in (a) 2022 and (b) 2023. Each color represents a different species that was observed on the cameras and the size of each section represents an approximate number of individuals counted for each species.

**FIGURE 5:**
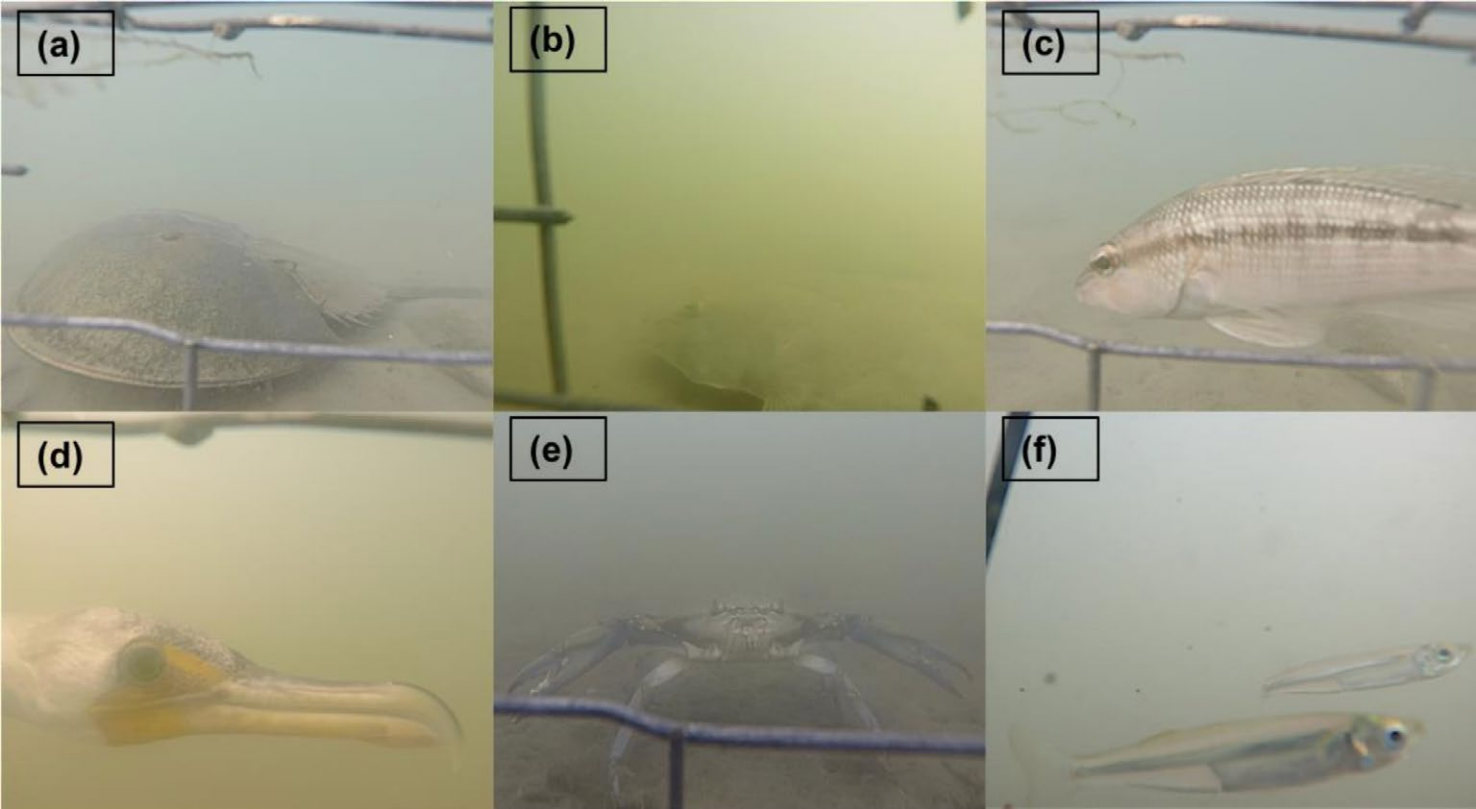
(a–f) Images collected from the underwater cameras that demonstrate some of the species that were observed and the quality of the camera footage. The species include (a) *L. polyphemus* , (b) *P. dentatus* , (c) *C. striata* , (d) *P. auritus* , (e) *C. sapidus* , and (f) *M. menidia* . These images were compiled from all monitoring sites.

Among these species, the greatest number of individuals and species recorded was at BBR. The site with the next highest number of recorded species was RBOC, both of these sites located on the east side of the Bay. The count of individuals of each species was estimated through the camera footage. The most abundant species recorded were *L. xanthurus* , *M. menidia* , *B. tyrannus* , *L. polyphemus* , *C. sapidus* , and *P. longicarpus* . The largest number of individuals of a single species was *B. tyrannus* and *M. menidia* . Overall, more species were detected at the pilot artificial reefs and private aquaculture farms compared to the control site (Figure 4). The underwater cameras were also able to record certain feeding behaviors and interactions of the species (Figure 5).

### 3.2 Identification of Targeted Species Using Real‐Time PCR for eDNA Analysis

**FIGURE 6:**
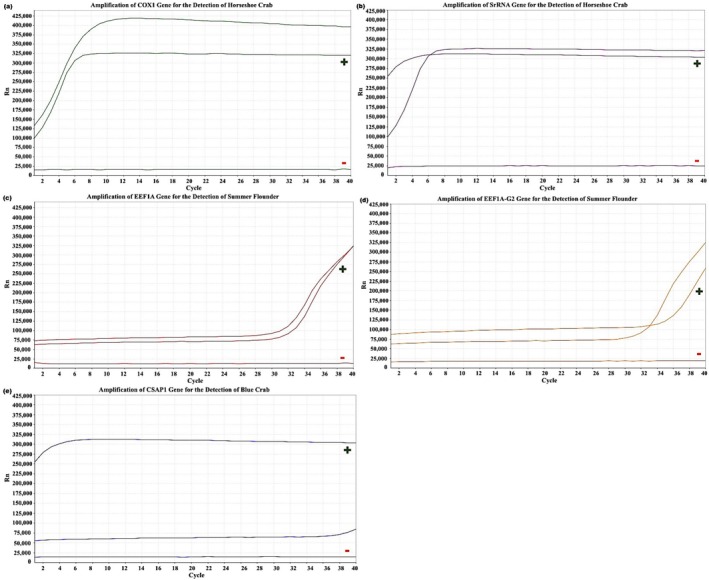
(a–e) Amplification of primers using qPCR. (a) COX1 and (b) SrRNA for the detection of *L. polyphemus* , (c) eef1a and (d) eef1a‐g2 for the detection of *P. dentatus* , and (e) Csap1 for the detection of *C. sapidus* with controls from BBR in 2023. Green (+) represent positive control samples and red (−) represent negative controls.

**FIGURE 7:**
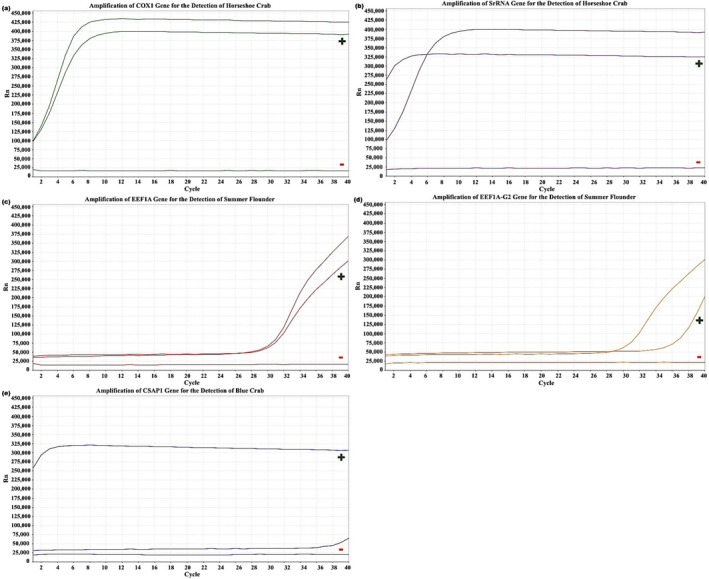
(a–e) Amplification of primers using qPCR. (a) COX1 and (b) SrRNA for the detection of *L. polyphemus* , (c) eef1a and (d) eef1a‐g2 for the detection of *P. dentatus* , and (e) Csap1 for the detection of *C. sapidus* with controls from CAH in 2023. Green (+) represent positive control samples and red (−) represent negative controls.

**FIGURE 8:**
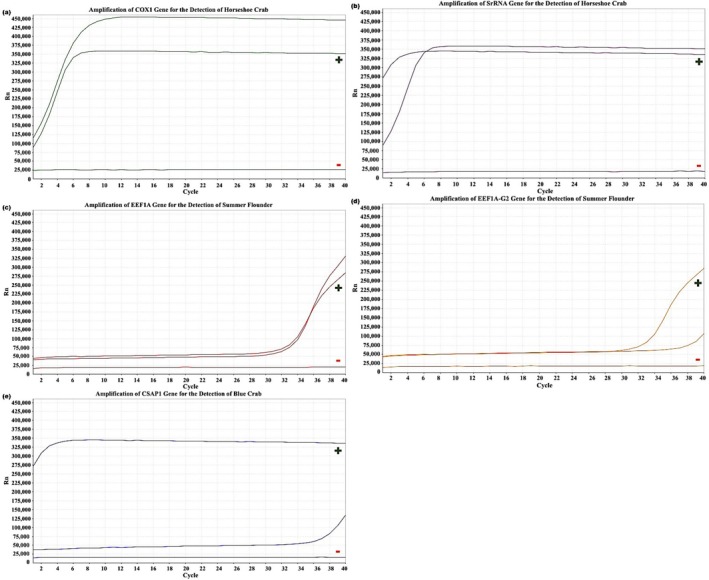
(a–e) Amplification of primers using qPCR. (a) COX1 and (b) SrRNA for the detection of *L. polyphemus* , (c) eef1a and (d) eef1a‐g2 for the detection of *P. dentatus* , and (e) Csap1 for the detection of *C. sapidus* with controls from RBOC in 2023. Green (+) represent positive control samples and red (−) represent negative controls.

**FIGURE 9:**
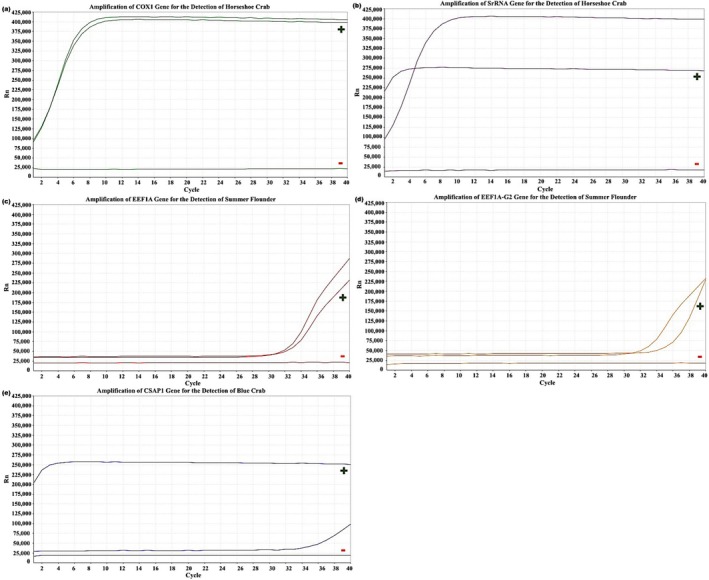
(a–e) Amplification of primers using qPCR. (a) COX1 and (b) SrRNA for the detection of *L. polyphemus* , (c) eef1a and (d) eef1a‐g2 for the detection of *P. dentatus* , and (e) Csap1 for the detection of *C. sapidus* with controls from SC in 2023. Green (+) represent positive control samples and red (−) represent negative controls.

**FIGURE 10:**
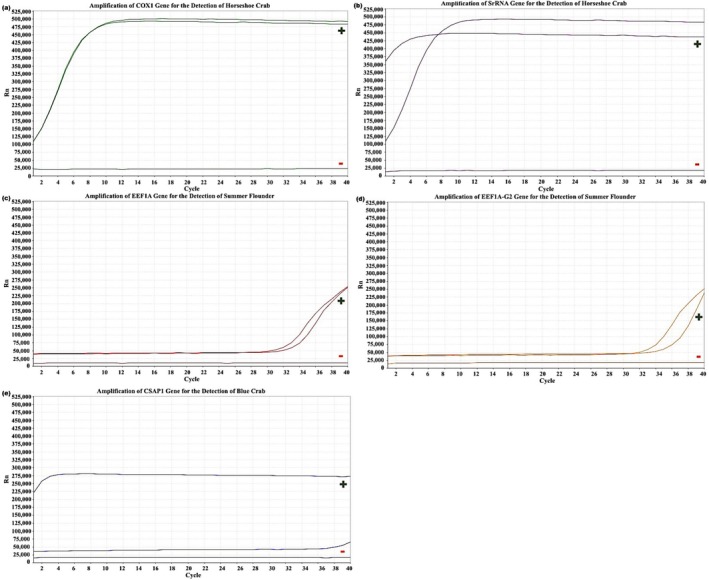
(a–e) Amplification of primers using qPCR. (a) COX1 and (b) SrRNA for the detection of *L. polyphemus* , (c) eef1a and (d) eef1a‐g2 for the detection of *P. dentatus* , and (e) Csap1 for the detection of *C. sapidus* with controls from SCC in 2023. Green (+) represent positive control samples and red (−) represent negative controls.

Samples were collected and analyzed from each of the five monitored locations in 2023 (Figures 6, 7, 8, 9, 10). The eDNA analysis using qPCR successfully detected *L. polyphemus* and *P. dentatus* across the sampling sites. Both targeted gene regions for *L. polyphemus* (COX1 and SrRNA) and *P. dentatus* (eef1a and eef1a‐g2) were successfully amplified, demonstrating the effectiveness of these markers in environmental monitoring applications. For *P. dentatus* , the eef1a‐g2 gene was detected at all sites except RBOC, while the eef1a gene was amplified consistently at all five sites. These findings suggest that while both gene targets are suitable for detection, the eef1a gene may offer slightly greater sensitivity or robustness under variable environmental conditions. Interestingly, *P. dentatus* was only visually observed at BBR via real‐time monitoring. This discrepancy between molecular and visual detection highlights the utility of eDNA approaches in capturing cryptic, mobile, or low‐abundance species that may be missed by conventional survey methods. It also emphasizes the need for cautious interpretation of absence data in visual surveys. Similarly, *L. polyphemus* was detected by qPCR at a majority of the sites and corroborated by real‐time visual observations. The consistency between molecular and visual detection for *L. polyphemus* strengthens confidence in the applied methodologies and supports the use of dual‐marker strategies to reduce false negatives and increase specificity. In contrast, attempts to detect *C. sapidus* using the Csap1 gene marker were unsuccessful at all sites, despite the species being clearly documented via underwater cameras. This inconsistency suggests several possible explanations: (1) the Csap1 primer set may have low sensitivity in environmental samples due to low gene copy number, poor primer‐template binding efficiency, or secondary structure interference; (2) environmental degradation of *C. sapidus* DNA may have occurred more rapidly than that of the other species, possibly due to behavior, physiology, or site‐specific environmental conditions; or (3) DNA extraction or PCR inhibition may have played a role, though this is less likely given the successful amplification of other species' markers in the same samples.

## 4 Discussion

Real‐time monitoring gave us a better understanding of species composition and distribution between the east and west sides of Rehoboth Bay, USA and between the different site types. We recorded several well‐recognized species that are abundant throughout the Bays, as well as some unique species such as the *C. ocellatus* , which is a tropical species. We were also able to observe certain feeding behaviors and interactions of species. Some of the notable interactions included a *P. auritus* diving for fish, a *R. bonasus* feeding on a *C. sapidus* , *C. sapidus* crawling along oyster cages, and fish feeding along the sediment floor. A majority of these interactions were observed at the aquaculture farms which demonstrate the habitat suitability of these sites. Both sites located on the east side of the Bay had greater species richness. This could be attributed to the east side's proximity to the Atlantic Ocean and the higher tidal exchange. Also, during storms or severe weather events, the east side of the Bay is more sheltered compared to the west side due to wind direction. It is important to note that the east side of the Bay also had greater camera visibility due to less turbid water conditions which is an influencing factor that needs to be considered for future studies. The site with the lowest number of diverse species was at the control site, SCC. It is evident that sites with some sort of habitat structure, in this case oyster reefs and aquaculture gear, have an increase in species richness and overall biodiversity. There were certain limitations with available camera equipment and since camera deployments were dependent on weather conditions and tidal influence, additional implementation of cameras is necessary to expand on biodiversity studies within Rehoboth Bay, Delaware, USA.

In this study, qPCR was successful in amplifying certain genes of our targeted species using eDNA from water samples. However, there were species that were observed through real‐time monitoring but were not detected in the eDNA analysis. These findings underscore the importance of ongoing marker validation and optimization for eDNA applications. The lack of amplification for *C. sapidus* despite confirmed presence by visual methods warrants a reevaluation of the Csap1 primer design and potentially the development or adoption of alternative genetic markers. Future work should include primer redesign using more conserved and high‐copy target regions (e.g., mitochondrial genes), validation on tissue‐derived DNA, and controlled spiking experiments to assess the limit of detection and amplification efficiency in complex environmental matrices. Overall, this study illustrates the strengths and limitations of eDNA‐based species detection for environmental monitoring in estuarine and coastal ecosystems. While molecular tools offer enhanced sensitivity and temporal resolution, careful calibration against physical survey data remains essential to ensure reliable biodiversity assessments and monitoring outcomes.

## 5 Conclusion

The use of both real‐time monitoring and eDNA analysis was an effective approach to monitoring biodiversity in the aquatic environment. As this research continues, the use of additional cameras can allow for monitoring of sites simultaneously and can provide a broader field of view. There is a further need to explore the capabilities of eDNA as a tool for biomonitoring. In the future, additional species will be included in the eDNA analysis and samples will be sent for eDNA metabarcoding to broaden the scope of species detection and for additional accuracy. The goal of this project was to assess the compatibility of both real‐time monitoring and eDNA analysis, while also collecting data that aims to promote sustainable aquaculture and continued oyster restoration efforts within the DIB. Understanding the role that *C. virginica* has in promoting biodiversity is essential to conserving coastal resiliency.

## Author Contributions

**Tahera Attarwala:** data curation (lead), formal analysis (lead), writing – original draft (lead). **Ali Parsaeimehr:** methodology (equal), validation (equal), writing – review and editing (equal). **Gulnihal Ozbay:** conceptualization (lead), funding acquisition (lead), project administration (lead), supervision (lead).

## Conflicts of Interest

The authors declare no conflicts of interest.

## Data Availability

The data that supports the findings of this study is openly available in OSF at https://osf.io/s8f7b/?view_only=ebadcc3441ef4a8f8d1e9d7fed9605c5.
